# Life-threatening danger assessments of penetrating injuries in Eastern Danish clinical forensic medicine

**DOI:** 10.1007/s00414-020-02485-9

**Published:** 2021-01-07

**Authors:** Lykke Schrøder Jakobsen, Marie Toftdahl Christensen, Sissel Banner Lundemose, Julie Munkholm, Anne Birgitte Dyhre Bugge, Niels Lynnerup, Jytte Banner

**Affiliations:** Department of Forensic Medicine, Section of Forensic Pathology, University of Copenhagen, Frederik V’s Vej 11, 2100 Copenhagen East, Denmark

**Keywords:** Clinical forensic medicine, Penetrating injury, Life-threatening danger assessment, Protocol implementation, Inter- and intra-rater agreement, Severity

## Abstract

Clinical forensic assessments of injuries’ life-threatening danger may have an impact on the legal aftermath following a violent assault. The pursuit of evidence-based guidelines should ensure a user-independent and reproducible forensic practice. However, does it? The aim of this study was to evaluate the forensic life-threatening danger assessments after a protocol implementation in 2016. The evaluation concerned usability and reproducibility of the protocol, and its influence on assessment severity. We analyzed the level of inter- and intra-rater agreement using 169 blinded, prior-protocol cases that were reassessed by two forensic specialists. We compared assessment made the year before and after protocol implementation (*n* = 262), and the forensic specialists’ reassessments with the prior-protocol cases’ original assessments (*n* = 169). Whether to make an assessment, the levels of agreement varied between weak agreement (inter-rater, *Κ* = 0.43; assessor 1, *Κ* = 0.57) and strong agreement (assessor 2, *Κ* = 0.90). Regarding severity, the levels of agreement varied between strong agreement (inter-rater, *Κ* = 0.87; assessor 1: *Κ* = 0.90) and almost perfect agreement (assessor 2: *Κ* = 0.94). The assessments were statistically significant redistributed after the implementation (chi-square test: *p* < 0.0001). The proportion of cases assessed as having not been in life-threatening danger increased from 9 to 43%, and moderate severity assessments decreased from 55 to 23%. Of the moderate severity assessments, 55% were reassessed as having not been in life-threatening danger. The protocol ensured independent and reproducible assessments when the forensic specialists agreed on making one. The protocol resulted in less severe assessments. Future studies should examine the reliability of the protocol and its consequences for legal aftermaths.

**Supplementary Information:**

The online version contains supplementary material available at 10.1007/s00414-020-02485-9.

## Introduction

### Forensic life-threatening danger assessments

The assessment of life-threatening danger or seriousness of documented injuries in clinical forensic medicine (CFM) is heterogeneous and lacks underlying evidence-based validation [1]. Studies discussing these types of assessments [2, 3] may not present their practice guidelines in detail or provide the underlying evidence-based validation, and this complicates any assessment of the scientific background or comparison of national practices. Life-threatening danger assessments are also part of the Danish standardized victim CFM examination [4], which is requested by the police authorities in cases of serious violence, as defined in the Danish Penal Code [5]. The CFM examination may contribute to the police investigation by clarifying some of the case circumstances, including the extent and dangerousness of the injuries. The latter may also have an impact on the decisions regarding the provisional charges by the police or the formal charges by the prosecution service against a suspected perpetrator [6].

### Implementation of a Danish protocol for assessment of life-threatening danger

The use of clinical guidelines has increased over many decades. These guidelines can facilitate the translation of medical research into recommendations, thereby closing the gap between daily practice and what is supported by a critical appraisal of scientific evidence, i.e., evidence-based guidelines [7]. Well-constructed and properly implemented guidelines should guide less experienced clinicians and convert experienced clinicians with outdated practices and should generally secure a standardized approach [7–9]. Expert opinions are not evidence, but they are of the utmost importance when developing guidelines [10].

**Fig. 1:**
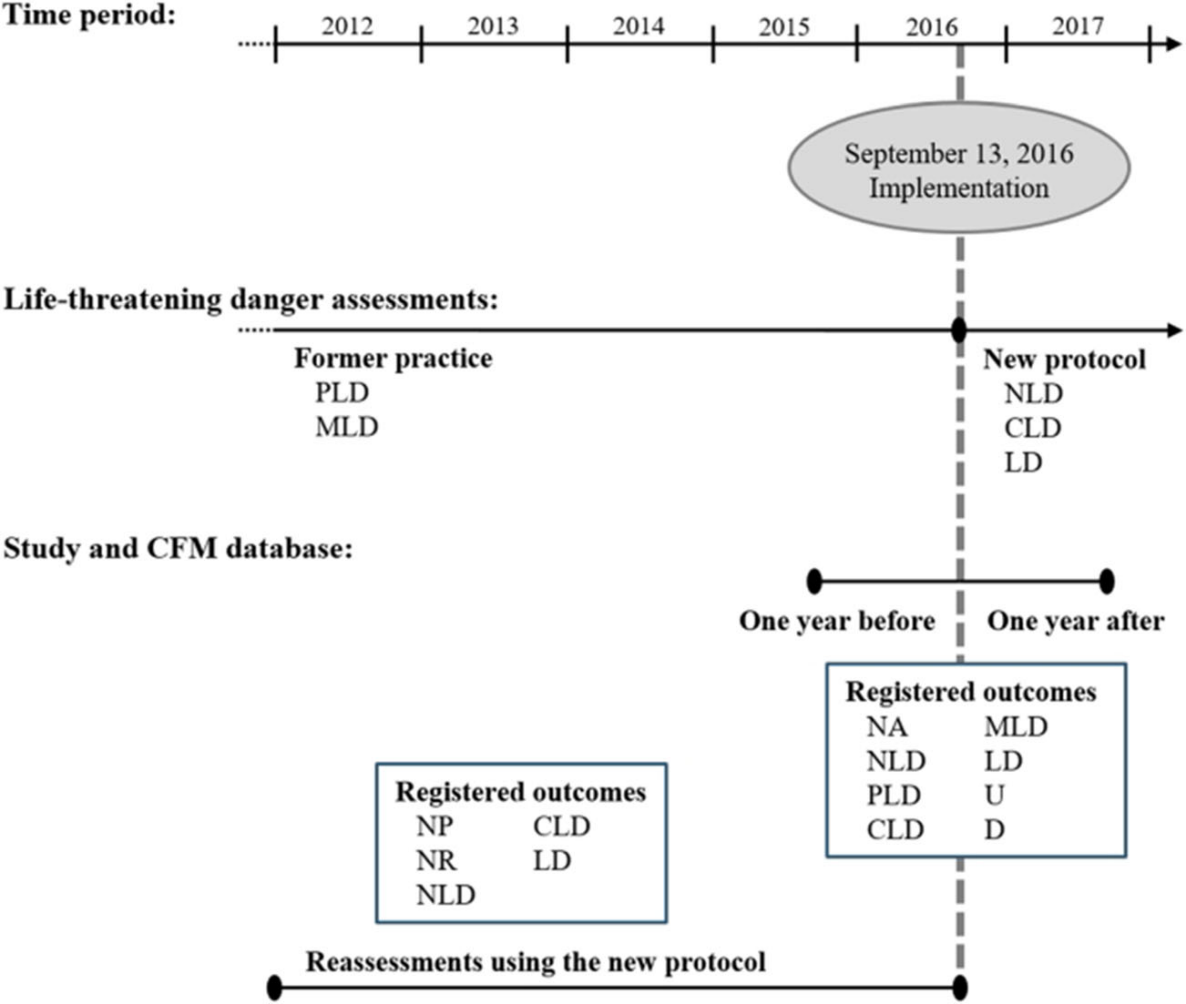
Time period, protocol implementation, and life-threatening danger assessments. One year before: from September 13, 2015, to September 12, 2016. One year after: from September 13, 2016, to September 12, 2017. Life-threatening danger assessments: not assessed (NA), not relevant (NR), not possible (NP), undetermined (U), has not been in life-threatening danger (NLD), potential life-threatening danger (PLD), could have been in life-threatening danger (CLD), manifest life-threatening danger (MLD), has been in life-threatening danger (LD), and died (D). For more information about the variables, methods, and analyses, see Table 1

On September 13, 2016, a revised local protocol was implemented as a result of a meeting between the Danish Medico-Legal Council and the Chief Forensic Pathologists in Denmark. This protocol is now used by the Department of Forensic Medicine, University of Copenhagen, in Eastern Denmark for life-threatening danger assessments [4]. Before the protocol implementation, the life-threatening danger assessments could be characterized as apprenticeship based on the expert opinions of the senior board-certified forensic specialists, and an educational textbook [11]. Apart from a terminology change (see Fig. 1 for an overview), the protocol specify that the Danish forensic life-threatening danger assessments should supersede the individual board-certified forensic specialists’ expert opinions and be based only on the documented, prior-to-treatment anatomical injuries and subsequent health state, and not on a hypothetical outcome of the violent act, i.e., tort-underlying argumentation. Thus, the local protocol should ensure inter-observer independent and reproducible life-threatening danger assessments, based on clinical-underlying argumentation. By removing the somewhat more legally grounded tort-underlying argumentation, this protocol could also be perceived as making the life-threatening danger assessments less severe.

Despite the potential conflict between the hospital personnel and the forensic specialists’ tasks, the cooperation works well and the forensic specialists in Eastern Denmark are mostly able to document the injuries before they are washed, stitched, and banded [4]. Information about the subsequent health state is available when the police, with a written consent from the examined individual, request hospital records from the healthcare sector [12]. The forensic specialists also document verbal communication with the hospital personnel during the CFM examinations. In cases with no objective signs of the assault, the argumentation that underlies the life-threatening danger assessment may lean on reported fatal outcomes in the scientific literature. This we refer to as literary-underlying argumentation.

The revised, local protocol includes a subdivision for penetrating injuries (i.e., sharp force injuries and gunshot wounds), with highlighted clinical-underlying criteria for the three possible life-threatening danger assessments. “Has not been in life-threatening danger” (NLD) is used when the CFM examined that the individual had stable vital parameters, sparse hemorrhage, no blood transfusion, no treatment except suturing, etc., whereas “could have been in life-threatening danger” (CLD) is used in cases with injuries that necessitated treatment, and “has been in life-threatening danger” (LD) is used when emergency treatment, surgery, blood transfusion, etc. were required. The abbreviations are listed in Table 1, and the subdivision of the protocol concerning the assessments of penetrating injuries is presented in English in Online Resource 1.

**Table 1:** Abbreviations, registered variables and possible entries, and methods and analyses

Life-threatening danger abbreviations			
List in alphabetical order			
CLD	Could have been in life-threatening danger	NLD	Has not been in life-threatening danger
D	Died	NP	Not possible
LD	Has been in life-threatening danger	NR	Not relevant
MLD	Manifest life-threatening danger	PLD	Potential life-threatening danger
NA	Not assessed	U	Undetermined
Methods and analyses			
Research question 1: Is the implemented protocol inter-observer independent and reproducible?			
Variables	Comparisons	Entries	Statistics
Reassessments	Yes/no danger assessment	NP + NR versus NLD + CLD + LD	Cohen’s kappa and McNemar’s test
Reassessments	Danger assessment’s severity	NLD versus CLD versus LD	Weighted kappa and Bowker’s test
Research question 2: Did the new protocol influence the severity of the life-threatening danger assessments compared to the former practice?			
Variables	Comparisons	Entries	Statistics
Original assessments	One year before and after protocol implementation	NA/NLD/PLD + CLD/MDL + LD/U/D	Two-way chi-square test
Original assessments and reassessments	Influence on severity	NA/NLD/PLD/CLD/MDL/LD/U/D versus NP/NR/NLD/CLD/LD	Descriptive

^a^Mutually exclusive with the other argumentation variables. Clinical-underlying argumentation, prior-to-treatment anatomical injuries and subsequent health state; literary-underlying argumentation, reported fatal outcomes in the scientific literature; tort-underlying argumentation, hypothetical outcome of the violent act

### Aim

The implementation of the revised local protocol raises two important questions: Given that the Eastern Danish forensic specialists adopted the new protocol, did it result in inter-observer independent and reproducible assessments, and did the protocol influence the severity of the life-threatening danger assessments of penetrating injuries? Therefore, the aim of this study was to evaluate the changed forensic practice regarding the life-threatening danger assessments of penetrating injuries in Eastern Danish forensics in terms of the usability and reproducibility of this subdivision of the new protocol and its influence on the assessment severity.

## Materials and methods

From a digital forensic case management system, we registered 3031 forensically examined individuals in Eastern Denmark from January 1, 2012, to December 31, 2017, in a CFM database. Inclusion criteria for the CFM database were CFM examinations of living individuals aged 15 or older, examined after a physical and/or sexual assault, from the geographical area, and in the time span defined above. Exclusion criteria were other kinds of forensic examinations (e.g., age evaluations, torture cases, individuals sampled solely for biological materials, and cases where the forensic reports were canceled or missing). As we evaluate the subdivision of the implemented protocol concerning penetrating injuries, we included only victim cases with documented sharp force injuries and gunshot wounds.

### Registered variables and statistics

When perusing the forensic reports, we registered information about the examination type, documented injuries, available hospital records, verbal communication with the hospital personnel from the healthcare sector, and, finally, the life-threatening danger assessment and its basis. Statistics were performed in SAS (SAS Enterprise Guide 7.15, 2017, SAS Institute Inc., Cary, NC, USA). The specific statistical analyses used are presented below. Figure 1 and Table 1 summarize the variables, possible entries, and the statistical analyses used.

### Articulated research questions

Using the following research questions, we evaluated the effect of the protocol implementation:

1. Is the implemented protocol inter-observer independent and reproducible?
2. Did the new protocol influence the severity of the life-threatening danger assessments compared to the former practice?

### Research question 1: Is the implemented protocol inter-observer independent and reproducible?

We selected 169 prior-protocol victim cases with documented penetrating injuries from the CFM database, 100 randomly selected sharp force injury cases [13], and all cases with gunshot wounds. Two board-certified forensic specialists (assessors 1 and 2) reassessed the 169 cases (see Fig. 1). After a minimum 2-month interval, they repeated the reassessment in 59 of the cases, which were randomly selected [13]. The reassessment process was time-consuming with approximately four reassessments per hour. Thus, we included 25% of the CFM examined victims with sharp force injuries (*N* = 396 cases in total). When reassessing the life-threatening danger, the assessors had the relevant original case material available (i.e., the anamnesis, objective forensic examination, obtained hospital records, and police report). We removed the original forensic report conclusion concerning the life-threatening danger assessment. For the inter-rater agreement analyses, we provided the assessors with a batch of approximately 60 cases each time. Possible reassessments were a priori defined as not possible (NP), not relevant (NR), NLD, CLD, and LD (see Fig. 1 and Table 1). The assessors were instructed to use NR when they found that the documented penetrating injuries were under a subjective lower threshold limit and/or a life-threatening danger assessment was not relevant for the case.

We tested the inter-rater agreement of the reassessments between assessors 1 and 2, and the intra-rater agreement (i.e., the test-retest reliability) of the reassessment for each assessor. We did not subdivide the statistical analyses based on the two types of penetrating injuries as the clinical-underlying criteria for the life-threatening danger assessments are the same for both sharp force injuries and gunshot wounds. Thus, we did not expect a reassessment difference between the two types of penetrating injuries.

The inter- and intra-rater analyses were a two-step process. Using Cohen’s kappa [14] and McNemar’s test [15], we tested the agreement of whether to make a life-threatening danger assessment (i.e., NP + NR versus NLD + CLD + LD). Second, we used weighted kappa [16, 17] and Bowker’s test of symmetry [18] to test the agreement of the life-threatening danger assessment severity (i.e., NLD versus CLD versus LD). Instead of using the SAS default Cicchetti-Allison linear weights [19], we chose to use the Fleiss-Cohen quadratic weights [16]. Thus, for the weighted kappa analyses, we used the AGREE(WT=FC) in the table statement in SAS [20]. We used McHugh’s interpretation of the kappa values for the level of agreement: 0–0.20 none, 0.21–0.39 minimal, 0.40–0.59 weak, 0.60–0.79 moderate, 0.80–0.90 strong, and above 0.90 almost perfect [21].

### Research question 2: Did the new protocol influence the severity of the life-threatening danger assessments compared to the former practice?

For this part of the study, we identified 262 forensically examined victims with penetrating injuries from September 13, 2015, to September 12, 2017 (i.e., the examinations performed in the year before and after the protocol implementation date; see Fig. 1) in the CFM database. We registered the availability of hospital records from the healthcare sector, verbal communication with the hospital personnel during the CFM examination, and the argumentation underlying the forensic life-threatening danger assessments.

#### Original life-threatening danger assessments 1 year before and after the protocol implementation

We compared the proportions of the cases with and without life-threatening danger assessments 1 year before and 1 year after the protocol implementation. Using a two-way chi-square test, we tested for a statistically significant distribution of the multinomial original life-threatening danger assessments between the two groups of our independent variable (i.e., the year before and the year after). In the chi-square test, we merged PLD and CLD and MLD and LD, to account for the terminology change (see Table 1).

#### Comparison of the original life-threatening danger assessments and reassessment severity

In order to examine whether the severity of the original life-threatening danger assessments would have differed had the protocol existed at the time of the CFM examinations, we compared the assessors’ 169 prior-protocol reassessed cases with the original life-threatening danger assessments. As the assessors were instructed to reassess the included cases, NA was not an option. Instead, they could state that it was not possible (NP) or not relevant (NR) to make a life-threatening danger assessment (see Fig. 1 and Table 1). We used descriptive statistics to compare the original life-threatening danger assessments and the reassessments.

## Results

### Research question 1: Is the implemented protocol inter-observer independent and reproducible?

The original life-threatening danger assessments for the 100 randomly selected victim cases with sharp force injuries were 24 NAs, 2 NLDs, 57 PLDs, 0 CLDs, 13 MLDs, 2 LDs, and 2 Ds. The distribution of the included victim cases with gunshot wounds (*N* = 67) was 10 NAs, 2 NLDs, 36 PLDs, 2 CLDs, 10 MLDs, 5 LDs, and 2 Ds. Two PLD cases had both documented sharp force injuries and gunshot wounds.

#### Inter-rater agreement

Comparison of the reassessments made by assessors 1 and 2 revealed agreement in 72% of the cases (Table 2). Table 2 shows that Cohen’s kappa for the inter-rater agreement analysis on whether to make a life-threatening danger assessment (NLD, CLD, and LD) or not (NP and NR) was 0.43 (95% CI, 0.23 to 0.62; *p* < 0.0001). According to McHugh, this is a weak level of agreement (0.40–0.59). The McNemar’s test was not statistically significant (*p* = 0.2008); therefore, we cannot reject that the reassessments made by assessor 1 and assessor 2 had equal distributions. The weighted kappa coefficient for the 136 included cases for the inter-rater severity agreement analysis was 0.87 (95% CI, 0.82 to 0.93; *p* < 0.0001). According to McHugh, this is a strong level of agreement (0.80–0.90). Bowker’s test of symmetry was statistically significant (*p* = 0.0062); therefore, we can reject that the raters had the same propensity to assess the degree of severity of the injuries. See Table 3.

**Table 2:** Reassessments of life-threatening danger by assessors 1 and 2

		Assessor 2					
		NP	NR	NLD	CLD	LD	Total
Assessor 1	NP	**7**	0	4	2	1	14
	NR	0	**4**	6	1	0	11
	NLD	5	2	**59**	5	1	72
	CLD	1	0	4	**13**	14	32
	LD	0	0	0	1	**39**	40
	Total	13	6	73	22	55	**169**

Assessor 1 and assessor 2 agreed in 122 out of 169 reassessed cases (72%). The cases where the assessors agreed and the total number of reassessments are highlighted in bold. See Table 1 for abbreviations

**Table 3:** Inter- and intra-rater agreement analyses for assessors 1 and 2

Research question 1: yes/no danger assessment								
		McNemar’s test			Cohen’s kappa			
	*N*	Chi^2^	DF	*p*	*Κ*	95% CL		*p*
Inter-rater	169	1.636	1	0.201	0.427	0.229	0.625	< 0.0001*
Intra-rater								
Assessor 1	59	0.667	1	0.414	0.567	0.259	0.875	< 0.0001*
Assessor 2	59	1.000	1	0.317	0.900	0.706	1.000	< 0.0001*
Research question 2: danger assessment severity								
		Bowker’s test			Quadratic weighted kappa			
	*N*	Chi^2^	DF	*p*	*Κ*	95% CL		*p*
Inter-rater	136	12.378	3	0.006*	0.872	0.817	0.927	< 0.0001*
Intra-rater								
Assessor 1	48	3.800	3	0.284	0.896	0.818	0.974	< 0.0001*
Assessor 2	53	2.000	3	0.572	0.942	0.892	0.992	< 0.0001*

McNemar’s and Bowker’s test H_0_, equal distribution of assessments between assessors 1 and 2. Cohen’s and weighted kappa H_0_, observed agreement due to chance (*Κ* = 0). A *p* < 0.05 was considered statistically significant (*). Chi-square test *p* value, PR > Chi^2^; and kappa test *p* value, two-sided Pr > |Z|

#### Intra-rater agreement

Regarding making a life-threatening danger assessment, the Cohen’s kappa coefficients for assessors 1 and 2 were 0.57 (95% CI, 0.26 to 0.88; *p* < 0.0001) and 0.90 (95% CI, 0.71 to 1.00; *p* < 0.0001), respectively. The McHugh’s interpretation is the same as for the inter-rater agreement analyses. The McNemar’s tests were both statistically non-significant (*p* = 0.4142 and *p* = 0.3173); thus, we cannot reject that the distributions of the reassessments were equal between the first and second reassessment. The weighted kappa values of the life-threatening danger assessment severity for assessor 1 and assessor 2 were 0.90 (95% CI, 0.82 to 0.97), *p* < 0.0001, and 0.94 (95% CI, 0.89 to 0.99), respectively. According to McHugh, the latter is interpreted as an almost perfect level of agreement. Bowker’s tests of symmetry were both not statistically significant (*p* = 0.2839 and *p* = 0.5724), so we cannot reject that the assessors had the same propensity to assess the degree of severity during the first and second time of reassessment (see Table 3).

### Research question 2: Did the new protocol influence the severity of the life-threatening danger assessments compared to the former practice?

Of the 262 included cases in the 2-year period, 201 had sharp force injuries, and 60 had gunshot wounds. There was one case with both documented sharp force injuries and gunshot wounds, as the second case with injury co-occurrence was from before September 13, 2015. The median age for the 53 included females was 23 years (15–76 years) and for the 209 included males 26 years (15–82 years). The life-threatening danger assessment was undetermined (U) in two cases, and five individuals died (D) before the forensic report was sent to the police. In total, 217 cases had a life-threatening danger assessment, and the proportion of these cases with registered hospital record and/or verbal communication increased from 89 to 93%. After the implementation, the number of clinical-underlying argumentations shows a statistically significant increase from 36 to 110 (chi-square = 18.92; *p* < 0.0001), whereas tort- and literary-underlying argumentations decreased from 47 to zero and from four to two, respectively. The year after implementation, 83% of the cases without an underlying danger assessment argument were NLD cases.

#### Original life-threatening danger assessments 1 year before and after the protocol implementation

Table 4 shows the number of cases with or without a life-threatening danger assessment the year before and after the implementation date of September 13, 2016. The proportion of NA cases was 13% the year before and 16% the year after the implementation. The PLD and MLD conclusions did not appear after the implementation. The proportion of cases with an NLD assessment increased from 9 to 43%. Conversely, the proportion of cases with a PLD or CLD assessment decreased from 55 to 23%. The proportion of cases with an MLD or LD assessment decreased from 20 to 17%. A Fisher’s exact test was performed between the life-threatening danger assessments made the year before and after the protocol implementation (Table 5) because some of the assessments had under five expected observations. A statistically significant redistribution was observed for the life-threatening danger assessments the year after implementation compared to the danger assessment distribution the year prior to implementation (chi-square = 44.96; *p* < 0.0001), and the NLD and PLD + CLD conclusions contributed the most (see cell chi-square values in Table 5).

**Table 4:** Original life-threatening danger assessments 1 year before and after protocol implementation

	Original life-threatening danger assessment, *N* (%)						
	NA	NLD	PLD	CLD	MLD	LD	Total
Before	15 (13)	11 (9)	47 (40)	17 (15)	17 (15)	6 (5)	117
After	23 (16)	62 (43)	0 (0)	33 (23)	0 (0)	24 (17)	145
Total	38 (15)	73 (28)	47 (18)	50 (19)	17 (6)	30 (11)	262

The year before implementation, September, 13, 2015, to September 12, 2016; the year after implementation, September 13, 2016, to September 12, 2017. See Table 1 for abbreviations. The two undetermined (U) and five deaths (D) are not shown in the table. The life-threatening danger assessment percentages are the row percentages

**Table 5:** Cell chi-square values from the chi-square analyses

	Original life-threatening danger assessment							
	NA	NLD	PLD + CLD	MLD + LD	U	D	Chi^2^	*p*
Protocol implementation								
Before	0.23	14.31	9.88	0.19	0.01	0.26	44.96^a^	< 0.0001*
After	0.18	11.55	7.97	0.16	0.01	0.21		
Clinical argument								
Before	–	8.10	1.91	4.24	–	–	18.92	< 0.0001*
After	–	2.65	0.63	1.39	–	–		

The year before implementation, September 13, 2015, to September 12, 2016; the year after implementation, September 13, 2016, to September 12, 2017. See Table 1 for abbreviations. The cell chi-square shows the contribution to the chi-square size for each category. A *p* < 0.05 was considered statistically significant (*)

^a^Fisher’s exact test was used because of expected cell counts less than five

#### Comparison of the original life-threatening danger assessments and reassessment severity

Table 6 compares the original life-threatening danger assessments with the reassessments made by the assessors in order to answer research question 1. Assessor 1 stated that assessment of the life-threatening danger of the documented injuries was not possible (NP) or not relevant (NR) in 41% of the original NA cases. That percentage was 32% for assessor 2. In 47% and 53% of the NA cases, assessors 1 and 2, respectively, assessed the case as NLD. Both assessors reassessed some of the NA cases as LD, 12% for assessor 1 and 15% for assessor 2. Of the original PLD + CLD cases, 28% (assessor 1) and 23% (assessor 2) were reassessed as CLD, whereas 55% of them resulted in an NLD assessment, and 7% (assessor 1) and 16% (assessor 2) in a LD assessment. The original MLD + LD cases were reassessed as LD in 83% and 100% of the cases by assessors 1 and 2, respectively. Assessor 1 reassessed the rest of the MLD cases as CLD (17%).

**Table 6:** Board-certified forensic specialists’ reassessments compared to the original life-threatening danger assessments

		Original life-threatening danger assessments								
Reassessments		NA	NLD	PLD	CLD	MLD	LD	U	D	Total
Assessor 1	NP	5	1	8	0	0	0	–	0	14
	NR	9	0	2	0	0	0	–	0	11
	NLD	16	3	52	1	0	0	–	0	72
	CLD	0	0	26	1	5	0	–	0	32
	LD	4	0	7	0	18	7	–	4	40
	D	0	0	0	0	0	0	–	0	0
	Total	34	4	95	2	23	7	–	4	169
Assessor 2	NP	5	2	6	0	0	0	–	0	13
	NR	6	0	0	0	0	0	–	0	6
	NLD	18	2	51	2	0	0	–	0	73
	CLD	0	0	22	0	0	0	–	0	22
	LD	5	0	16	0	23	7	–	4	55
	D	0	0	0	0	0	0	–	0	0
	Total	34	4	95	2	23	7	–	4	169

See Table 1 for abbreviations

## Discussion

### Statements of principal findings

Our first main finding, based on our results, was that the protocol needs criteria for *when* it should be used. However, when the board-certified forensic specialists decided to make a life-threatening danger assessment, the severity of the danger assessments were inter-rater independent and reproducible. The McNemar’s test indicated that the evidence is insufficient to conclude that no agreement exists between assessors 1 and 2 (*p* = 0.2008), but the level of agreement on whether to make a life-threatening danger assessment was weak, with a Cohen’s kappa coefficient of 0.43. By contrast, the inter-rater severity, with a weighted kappa coefficient of 0.87 (95% CI, 0.82 to 0.93), can be interpreted as a strong level of agreement [21], despite a Bowker’s test of symmetry that was statistically significant (*p* = 0.0062), which means that the assessors did not have the same propensity to assess the severity of the injuries. The latter is consistent with the findings in Table 2, where assessor 1 had far more CLDs than assessor 2, and assessor 2 generally assessed the life-threatening danger as more severe than assessor 1. The same patterns regarding the degree of agreement were seen for assessor 1’s intra-rater agreement analyses (yes/no danger assessment, *Κ* = 0.57; and danger assessment’s severity, *Κ* = 0.90), whereas both of assessor 2’s kappa coefficients indicated a strong (yes/no danger assessment) and almost perfect (severity) degree of intra-rater agreement [21]. For both assessors, the tests regarding symmetry were not statistically significant (i.e., the evidence was insufficient to reject agreement and they both had the same propensities to assess the severity of the injuries).

Our second main finding was that the life-threatening danger assessments became less severe when the two board-certified forensic specialists reassessed prior-protocol cases. PLD and MLD did not appear after the protocol implementation, which indicates that the forensic specialists immediately adopted the new protocol. The increase in NLDs and the assessors’ reassessments of NA cases as NLD (47% and 53%, respectively) could indicate an increased post-protocol *active* mentioning of nonlife-threatening danger instead of a prior-protocol passive omission in cases where the examined victims were assessed as having not been in life-threatening danger. However, no increase was noted in the proportion of cases with a life-threatening danger assessment after the protocol implementation (82%) compared to the former practice (84%). Instead, the life-threatening danger assessments statistically significantly changed from PLD + CLD to NLD. The latter was also indicated by the assessors’ reassessments of prior-protocol cases that resulted in a severity decrease, as 55% of the prior-protocol PLD + CLD cases were reassessed as NLDs. The results indicate that most prior-protocol cases with a PLD + CLD conclusion would have been assessed as NLD had the protocol implementation happened earlier. This most likely is a result of the protocol’s outlined, clinical-underlying criteria and the resulting increase in clinical-underlying argumentation, as well as the exclusion of the hypothetical outcome of the violent act (i.e., the tort-underlying argumentation).

Both assessors reassessed prior-protocol NA cases as LD, which may indicate that some of the cases should have had a life-threatening danger assessment. In 57% of assessor 1’s and 31% of assessor 2’s NPs, they stated that it was not possible to reassess the life-threatening danger despite an available hospital record (data not shown). The CFM database’s lack of specification of “hospital record” (e.g., emergency records, ward round records, or operative notes) may explain their decision not to reassess the case as; in multiple cases, they stated that they specifically lacked an operation description. A future recommendation could be to specify what information should be available.

### Strength and limitations of this study

Random selection of sharp force injury cases for the reassessments should prevent selection bias in terms of case circumstances and the information upon which the life-threatening danger assessment was based (e.g., information of vital parameters, age, co-occurrence of injury types). Thus, they are a representative sample of cases with documented sharp force injuries. Blinding the original life-threatening danger assessments, the unknown distribution of them and the random selection of the cases for the batches ensured that the assessors could not predict the original assessments. Moreover, the 2-month interval between the first and second reassessments and the random selection of cases for the intra-observer agreement analyses ensured minimized recall bias. Lastly, it could be argued that the type of penetrating injury may have influenced the assessors as one could speculate that gunshot wounds were assessed more seriously than sharp force injuries. However, the protocol’s highlighted criteria comprise both types of penetrating injuries and should ensure that only the prior-to-treatment anatomical injuries and subsequent health state form the basis of the life-threatening danger assessment, not the type of injury.

The use of the Fleiss-Cohen quadratic weights [16] for the weighted kappa analyses resulted in a graduation of the disagreements, where the penalties for disagreement become severer as the disagreements become larger [17]. We consider this to be the most appropriate approach regarding the assessments of life-threatening danger severity.

The reassessments may not be representative for all the department’s board-certified forensic specialists who use the protocol due to the nonrandom selection of the assessors, who have special clinical forensic responsibilities. Moreover, the selected cases with penetrating injuries represent only one-third of the protocol, and our conclusions regarding the usability of the protocol are therefore restricted to cases with documented sharp force injuries and/or gunshot wounds.

We consider the inclusion of all forensic victim examinations in the 2-year period as a strength, but the selection of the year up to and just after the implementation may not be representative for the implementation effect in the longer term. We do not know whether an intensified focus on the new protocol occurred during the selected time interval. To consider this, we could have chosen, for example, the years 2013 and 2019.

The protocol is part of the department’s DANAK-accredited regulatory tasks [4, 22, 23] and is regularly updated. However, despite moving from individual expert opinions to a standardized approach and consistent terminology, the forensic life-threatening danger assessments lack evidence-based validation.

## Conclusion

Our results suggest that the protocol is inter-observer independent and reproducible when the board-certified forensic specialists agreed that a life-threatening danger assessment should be made. However, the results also suggest that the protocol must include and specify criteria for *when* it should be used. The protocol implementation did not result in an increased number of life-threatening danger assessments, but the changed terminology and the disappearance of the tort-underlying argumentation are indications of successful protocol implementation. Using the implemented protocol on prior-protocol cases made the life-threatening danger assessment less severe, as the number of post-protocol NLD cases increased and the majority of the prior-protocol, tort-argued PLD + CLD cases were reassessed as NLD by the board-certified forensic specialists.

### Perspective

The results raise some very important questions: How can we know that the new protocol is better than the former practice, and what impact do the changed forensic life-threatening danger assessments have on the legal aftermath? Evidence-based CFM is challenging, as it is not possible to perform randomized clinical trials, which is the pinnacle of the evidence hierarchy [10, 24, 25]. Despite the fact that the tort-underlying argumentation was replaced by the clinical-underlying argumentation, the evidence behind the implemented protocol is unknown. Thus, the protocol cannot be characterized as an evidence-based guideline. Therefore, future studies should examine the reliability of the protocol and its clinical-underlying argumentation, as well as the impact of forensic life-threatening danger assessments on the legal aftermath and the potential consequences of the documented severity decrease.

## Supplementary information

ESM 1 (PDF 320 kb)

## Data Availability

Not applicable.

## References

[1] Meilia PDI, Freeman MD, Herkutanto ZMP (2018). A review of the diversity in taxonomy, definitions, scope, and roles in forensic medicine: implications for evidence-based practice. Forensic science, medicine, and pathology.

[2] Gignon M, Paupiere S, Jarde O, Manaouil C (2010). Victims of assault: a Europe-wide review of procedures for evaluating the seriousness of injuries. Med Sci Law.

[3] Pfeiffer JL, Pueschel K, Seifert D (2016). Interpersonal violence in road rage. Cases from the Medico-Legal Center for victims of violence in Hamburg. Journal of Forensic and Legal Medicine.

[4] Jakobsen LS, Jacobsen C, Lynnerup N, Steinmetz J, Banner J (2020) Clinical forensic medicine in Eastern Denmark: organisation and assessments. Med Sci Law:25802419898338. doi:10.1177/0025802419898338 10.1177/0025802419898338 32090675

[5] Bekendtgørelse af straffeloven [Ministerial order of the Danish Penal Code] (2019). vol LBK nr 976 af 17/09/2019, j.nr. 2019–730-0330. Ministry of Justice, Copenhagen

[6] Danish Prosecution Service (n.d.) The criminal justice process. https://anklagemyndigheden.dk/en/report-and-investigation. Accessed 4 June 2019

[7] Woolf SH, Grol R, Hutchinson A, Eccles M, Grimshaw J (1999). Clinical guidelines: potential benefits, limitations, and harms of clinical guidelines. BMJ (Clinical research ed).

[8] Gundersen L (2000). The effect of clinical practice guidelines on variations in care. Ann Intern Med.

[9] Fischer F, Lange K, Klose K, Greiner W, Kraemer A (2016) Barriers and strategies in guideline implementation-a scoping review. Healthcare (Basel) 4(3). 10.3390/healthcare4030036 10.3390/healthcare4030036 PMC5041037 27417624

[10] Balshem H, Helfand M, Schunemann HJ, Oxman AD, Kunz R, Brozek J, Vist GE, Falck-Ytter Y, Meerpohl J, Norris S, Guyatt GH (2011). GRADE guidelines: 3. Rating the quality of evidence. J Clin Epidemiol.

[11] Simonsen J (1999). Klinisk retsmedicin. Voldsforbrydelser [Clinical forensic medicine. Violent crime]. Retsmedicin og medicinal lovgivning [Forensic and medical legislation].

[12] Bekendtgørelse af sundhedsloven [Ministerial order of the Danish Health Act] (2019). vol LBK nr 903 af 26/08/2019, j.nr. 1901998. Ministry of Health, Copenhagen

[13] Maple Tech. International LLC. (2020) Random number generator. https://www.calculator.net/random-number-generator.html. Accessed MISSING

[14] Lund Research Ltd (2018) Statistics Laerd, Cohen’s kappa. https://statistics.laerd.com/.

[15] Lund Research Ltd (2018) Statistics Laerd, McNemar’s test. https://statistics.laerd.com/.

[16] Fleiss JL, Cohen J (1973). The equivalence of weighted kappa and the intraclass correlation coefficient as measures of reliability. Educ Psychol Meas.

[17] Lund Research Ltd (2018) Statistics Laerd, Weighted kappa, types of weights: linear versus quadratic. https://statistics.laerd.com/.

[18] Bowker AH (1948). A test for symmetry in contingency tables. J Am Stat Assoc.

[19] Cicchetti DV, Allison T (1971). A new procedure for assessing reliability of scoring EEG sleep recordings. American Journal of EEG Technology.

[20] SAS Institute Inc. (2019) SAS visual statistics 8.4: procedures, the FREQTAB procedure, kappa weights. https://documentation.sas.com/?docsetId=casstat&docsetTarget=casstat_freqtab_details76.htm&docsetVersion=8.4&locale=en. Accessed 18 June 2020

[21] McHugh ML (2012). Interrater reliability: the kappa statistic. Biochem Med (Zagreb).

[22] Danish Accreditation Fund (DANAK) (n.d.) DANAK - Danish Accreditation Fund. http://english.danak.dk/.

[23] Danish Accreditation Fund (DANAK) (n.d.) 02-9042 Inspektion: Retspatologiske undersøgelser. http://published.danak.dk/register.asp?sag=02-9042&nohead=y.

[24] Atkins D, Best D, Briss PA, Eccles M, Falck-Ytter Y, Flottorp S, Guyatt GH, Harbour RT, Haugh MC, Henry D, Hill S, Jaeschke R, Leng G, Liberati A, Magrini N, Mason J, Middleton P, Mrukowicz J, O'Connell D, Oxman AD, Phillips B, Schünemann HJ, Edejer T, Varonen H, Vist GE, Williams JW, Zaza S (2004). Grading quality of evidence and strength of recommendations. BMJ (Clinical research ed).

[25] Guyatt GH, Oxman AD, Vist GE, Kunz R, Falck-Ytter Y, Alonso-Coello P, Schünemann HJ (2008). GRADE: an emerging consensus on rating quality of evidence and strength of recommendations. BMJ (Clinical research ed).

