# Supplementary material for: Life-threatening danger assessments of penetrating injuries in Eastern Danish clinical forensic medicine
Source: Int J Legal Med. 2021 Jan 7;135(3):861–70. doi: 10.1007/s00414-020-02485-9 (PMC8036202; doi:10.1007/s00414-020-02485-9)
Supplement: Supplementary file 1 — (PDF 320 kb) [file 414_2020_2485_MOESM1_ESM.pdf]

Electronic Supplementary Material (Online Resource)

Eastern Danish protocol regarding assessment of life-threatening danger assessment

Article

Life-threatening danger assessments of penetrating injuries in Eastern Danish clinical forensic medicine

Journal

International Journal of Legal Medicine

Authors

Lykke Schrøder Jakobsen[1]

Marie Toftdahl Christensen[1]

Sissel Banner Lundemose[1]

Julie Munkholm[1]

Anne Birgitte Dyhre Bugge[1]

Niels Lynnerup[1]

Jytte Banner[1]

Affiliation

[1] Department of Forensic Medicine, University of Copenhagen, Denmark

University of Copenhagen, Department of Forensic Medicine, Section of Forensic Pathology.

Frederik V's Vej 11, 2100 Copenhagen East, Denmark.

E-mail address of the corresponding author

Lykke Schrøder Jakobsen: lykke.jakobsen@sund.ku.dk

Protocol regarding assessment of life-threatening danger during the CFM examination		
Version: X	Scope: Forensic specialists	Replace: Version X
Formulated by: [Name]<br>Reviewed by: [Name]<br>Date: [Date]	Approved by: [Name]<br>Date: [Date]	Take effect as from: [Date]

Aim: To ensure consensus regarding the life-threatening danger assessments of documented injuries during the CFM examination.

Overall principles

An assessments regarding life-threatening danger will be part of the CFM examination in the following types of violence:

- Non-fatal strangulation
- Penetrating injuries: Stabs, cuts, strokes, and gunshot wounds
- Severe blunt force violence against the head and body

For the last two types of violence, the assessment must relate to the documented injuries, not the violent act itself or the situation, which the examined individual was in, when the violence occurred. The injury can only be assessed as being life-threatening, if there is a real possibility that the injury, untreated, would be fatal.

Statements regarding life-threatening danger

The statement regarding life-threatening danger is worded as follows:

- The examined individual has been in life-threatening danger, as...
- The examined individual could have been in life-threatening danger, as...
- The examined individual has not been in life-threatening danger, as...

The paragraph preceding the life-threatening danger assessment summarizes the documented and informed findings, which underlie the assessment. In case of violence against the neck, this paragraph also includes the examined victim’s symptoms. *The examples written in italics* in the table below are suggestions for the formulation of the documented and clinical findings.

Below are examples of different scenarios of the three types of violence and the associated wording regarding life-threatening danger. [Only penetrating injuries are presented in the following, ed.]

Penetrating injuries (Stabs, cuts, and gunshot wounds):	
**Emergency treatment required, operation, blood transfusion etc.**<br>Example: Lesion of the heart and larger vessels.	*The examined individual was unconscious/awake on arrival at the trauma center with low blood pressure and rapid pulse.*<br>*1500 ml of blood was given and the incision of the carotid artery was sutured.*<br><br>In the present case, it must be assumed that the examined individual <u>has been in life-threatening danger</u>, as the examined individual without the acute, competent medical treatment would not/hardly have survived.
**Stable vital parameters, sparse hemorrhage, no blood transfusion, but treatment with suturing etc.**<br>Example: Lesion of minor vessels, intestines and pneumothorax.	*The examined individual was awake on arrival at the trauma center with normal blood pressure and normal pulse.*<br>*Acute opening of the abdominal cavity was performed with suturing of a small lesion in the small intestine. No blood transfusion was given.*<br><br>In the present case, it must be assumed that the examined individual <u>could have been in life-threatening danger</u>, as the examined individual without the medical treatment could have been put in a state that he/she might not have survived.
**Stable vital parameters, sparse hemorrhage, no blood transfusion, no treatment except suturing etc.**<br>Example: Superficial stabs, cuts or tangential gunshot wounds.	*During the entire therapy, the examined individual was awake and with normal blood pressure and normal pulse. No treatment other than suturing of the superficial incision was given.*<br><br>In the present case, it must be assumed that the examined individual <u>has not been in life-threatening danger</u>, as he/she most likely would have survived without the medical treatment of the documented lesions.<br><br>or<br><br>*During the entire therapy, the examined individual was awake and with normal blood pressure and normal pulse. The examined individual was discharged without treatment of the documented bruises.*<br><br>In the present case, it must be assumed that the examined individual <u>has not been in life-threatening danger</u>, as the documented lesions did not necessitate treatment.

Overview of revisions

**Version number:**	**Take effect as from:**	**Must be reviewed no later than:**	**Reason for revision:**	**Signature:**
Version 1	[Date]	[Month, year]	New protocol	[Name]
Version 2	[Date]	[Month, year]	...	[Name]
Version ...	[Date]	[Month, year]	...	[Name]
